# Sulfur Assimilation Alters Flagellar Function and Modulates the Gene Expression Landscape of Serratia marcescens

**DOI:** 10.1128/mSystems.00285-19

**Published:** 2019-08-06

**Authors:** Mark T. Anderson, Lindsay A. Mitchell, Anna Sintsova, Katherine A. Rice, Harry L. T. Mobley

**Affiliations:** a Department of Microbiology and Immunology, University of Michigan Medical School, Ann Arbor, Michigan, USA; DOE Joint Genome Institute

**Keywords:** *Serratia*, cysteine, flagella, hemolysin, phospholipase, sulfur

## Abstract

Sulfur is an essential nutrient that contributes to cellular redox homeostasis, transcriptional regulation, and translation initiation when incorporated into different biomolecules. Transport and reduction of extracellular sulfate followed by cysteine biosynthesis is a major pathway of bacterial sulfur assimilation. For the opportunistic pathogen Serratia marcescens, function of the cysteine biosynthesis pathway is required for extracellular phospholipase activity and flagellum-mediated surface motility, but little else is known about the influence of sulfur assimilation on the physiology of this organism. In this work, it was determined that an S. marcescens cysteine auxotroph fails to differentiate into hyperflagellated and elongated swarmer cells and that cysteine, but not other organic sulfur molecules, restores swarming motility to these bacteria. The S. marcescens cysteine auxotroph further exhibits reduced transcription of phospholipase, hemolysin, and flagellin genes, each of which is subject to transcriptional control by the flagellar regulatory system. Based on these data and the central role of cysteine in sulfur assimilation, it was reasoned that environmental sulfur availability may contribute to the regulation of these functions in S. marcescens. Indeed, bacteria that are starved for sulfate exhibit substantially reduced transcription of the genes for hemolysin, phospholipase, and the FlhD flagellar master regulator. A global transcriptomic analysis further defined a large set of S. marcescens genes that are responsive to extracellular sulfate availability, including genes that encode membrane transport, nutrient utilization, and metabolism functions. Finally, sulfate availability was demonstrated to alter S. marcescens cytolytic activity, suggesting that sulfate assimilation may impact the virulence of this organism.

**IMPORTANCE** Serratia marcescens is a versatile bacterial species that inhabits diverse environmental niches and is capable of pathogenic interactions with host organisms ranging from insects to humans. This report demonstrates for the first time the extensive impacts that environmental sulfate availability and cysteine biosynthesis have on the transcriptome of S. marcescens. The finding that greater than 1,000 S. marcescens genes are differentially expressed depending on sulfate availability suggests that sulfur abundance is a crucial factor that controls the physiology of this organism. Furthermore, the high relative expression levels for the putative virulence factors flagella, phospholipase, and hemolysin in the presence of sulfate suggests that a sulfur-rich host environment could contribute to the transcription of these genes during infection.

## Abstract (precis)

Serratia marcescens is a versatile bacterial species that inhabits diverse environmental niches and is capable of pathogenic interactions with host organisms ranging from insects to humans. This report demonstrates for the first time the extensive impacts that environmental sulfate availability and cysteine biosynthesis have on the transcriptome of S. marcescens. The finding that greater than 1,000 S. marcescens genes are differentially expressed depending on sulfate availability suggests that sulfur abundance is a crucial factor that controls the physiology of this organism. Furthermore, the high relative expression levels for the putative virulence factors flagella, phospholipase, and hemolysin in the presence of sulfate suggests that a sulfur-rich host environment could contribute to the transcription of these genes during infection.

## INTRODUCTION

Bacteria continually adapt to their external environment in response to exogenous cues such as nutrient availability, antagonistic threats, temperature, and other signals. For bacterial species that are capable of inhabiting a broad range of environments, the sensing of changing conditions and the initiation of an appropriate physiologic response to a given environment are critical. Sulfur availability serves as an example of this type of bacterial interaction with the environment. Sulfur is an essential element that is bioavailable in both organic and inorganic forms and is incorporated into numerous biomolecules, such as amino acids (e.g., cysteine and methionine) and cellular cofactors (e.g., glutathione and biotin). The bacterial requirement for sulfur can be satisfied by multiple means, but a well-characterized and conserved mechanism of sulfur acquisition is through assimilation of the sulfate anion (1). Following transport, sulfate is reduced to sulfide and is incorporated into cysteine, which in turn serves as a central source of reduced sulfur for incorporation into other biomolecules.

**FIG 1:**
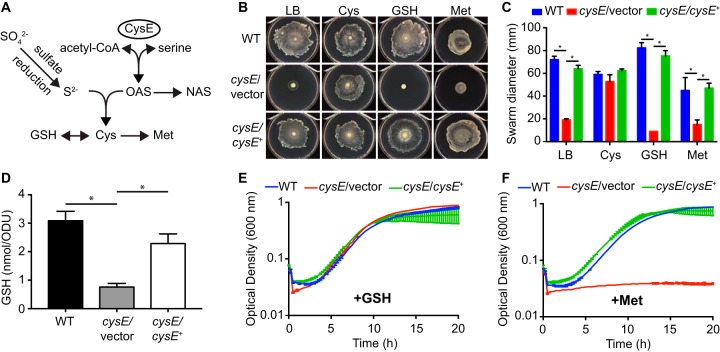
Cysteine limitation prevents swarming motility of S. marcescens. (A) Model of sulfate assimilation and cysteine biosynthesis derived from established pathways in E. coli. Intermediate steps in the glutathione (GSH), methionine, and sulfate reduction pathways are not depicted. (B) Representative images of swarming motility for S. marcescens strains on soft agar plates. Wild-type (WT), *cysE* mutant (*cysE*/vector), and the complemented mutant (*cysE*/*cysE*^+^) strains were spotted onto the surfaces of LB agar plates or LB agar supplemented with 1 mM cysteine, glutathione, or methionine and allowed to swarm for 16 h. (C) Quantitation of swarming motility. The swarm zone diameters were determined for triplicate cultures and the mean diameters (± standard deviations) are presented. *, *P* < 0.01 by *t* test. (D) Total intracellular glutathione from bacteria cultured in LB was measured for each strain and normalized to the culture optical density. Bars represent the means (± standard deviations) from triplicate cultures. *, *P* < 0.01 by *t* test. (E) Growth of S. marcescens strains in M9 medium supplemented with 1 mM glutathione. Growth was measured by optical density in 15-min intervals. Values represent the means (± standard deviations) from triplicate cultures. (F) Growth of S. marcescens strains in M9 medium supplemented with 1 mM methionine as described for panel E.

The pathways for sulfur transport and cysteine biosynthesis in Escherichia coli and Salmonella enterica serovar Typhimurium are regulated by at least two mechanisms (1, 2). First, activity of the serine acetyltransferase enzyme CysE is subject to cysteine-mediated feedback inhibition (3). Additional regulation is provided at the transcriptional level by the CysB protein, together with *N*-acetyl-l-serine (NAS) as an inducer (4), for genes encoding sulfur assimilation and cysteine biosynthesis functions. NAS is derived nonenzymatically from the CysE product *O*-acetyl-l-serine (OAS) in the first step of the cysteine biosynthesis pathway (Fig. 1A). Thus, when sulfur sources are limited, CysB and NAS activate transcription of genes encoding sulfate transport and reduction functions as well as cysteine biosynthesis enzymes, which are collectively referred to as the cysteine regulon (5, 6). Cysteine metabolism has also been observed to impact biofilm formation and carbon source utilization in multiple bacterial species (7–11) as well as siderophore and extracellular protease production in *Pseudomonas* (12).

Serratia marcescens is a member of the *Yersiniaceae* family of enterobacteria that inhabits both environmental and animal-associated niches. S. marcescens is an emerging cause of health care-associated infections that range from urinary tract infections to deadly bloodstream infections (13). Our previous work established that disruption of the *cysE* gene in S. marcescens resulted in reduced extracellular phospholipase activity and swarming motility (14). Importantly, these two processes both require flagellar function. PhlA phospholipase export is mediated by the flagellar secretion apparatus, and swarming motility requires differentiation into elongated and hyperflagellated cells (14–18). Additional work demonstrated that expression levels of *phlA* and the flagellar regulator genes *flhD* and *fliA* were decreased in *cysE* mutant bacteria (14). The *Serratia* FlhDC flagellar master regulator complex, together with the flagellar sigma factor FliA, are a central hub in a complex regulatory network that manages multiple regulatory inputs and controls expression of numerous genes (19–22). In addition to *phlA* (17, 23), of interest among the flagellar regulon genes is *shlA* (20, 21). The ShlA pore-forming hemolysin is an extracellular protein that contributes to the pathogenesis of S. marcescens in multiple model systems (20, 24–27). Consequently, environmental cues that impact the activity of the flagellar regulatory cascade have the potential to alter the physiology of S. marcescens, both through modulation of flagellar function and the pathogenic potential of the organism. The requirement for *cysE* in high-level expression of *flhD* and *fliA* suggests that the sulfur assimilation pathway may exert a regulatory influence on this process, in addition to the expected effect on cysteine regulon functions.

In this work, we investigated the process by which sulfur assimilation and cysteine biosynthesis alter global S. marcescens gene expression. We report that sulfate-starved bacteria exhibit decreased transcription of hemolysin, phospholipase, and flagellar genes and further define the sulfate-responsive regulon of S. marcescens using a transcriptomic approach. Our results additionally demonstrate that S. marcescens hemolytic activity and flagellar function are dependent on an intact cysteine biosynthesis pathway and the presence of sulfate.

## RESULTS

### Swarming motility and cellular differentiation require cysteine.

**FIG 2:**
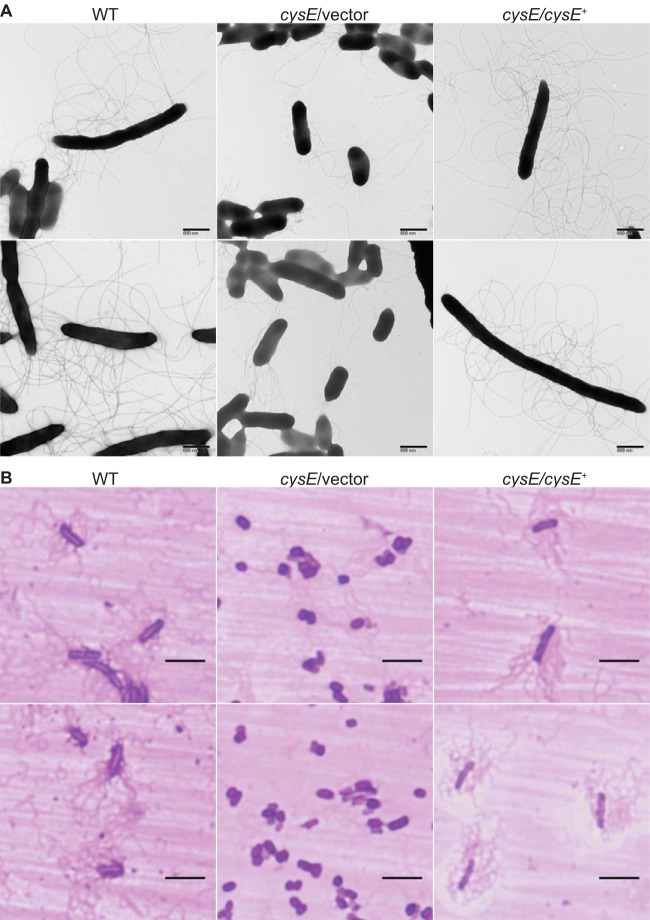
Cysteine-dependent swarm cell differentiation of S. marcescens. (A) Wild-type (WT), *cysE* mutant (*cysE*/vector), and complemented mutant (*cysE*/*cysE*^+^) strains were collected from the leading edges of actively growing swarm cultures and applied to grids for staining with 1% phosphotungstic acid. Bacterial cells were visualized by transmission electron microscopy, and representative images are shown. (B) Bacteria collected from swarm zones as described for panel A were stained for flagella on glass slides. Representative images of stained S. marcescens strains collected by light microscopy using a 100× lens objective are shown. Bars, 5 μm.

Surface inoculation of the S. marcescens UMH9 bacteremia isolate onto LB medium solidified with 0.6% agar resulted in the expansion of bacteria across the surface of the medium within 16 h (Fig. 1B). This surface motility was quantitated by measuring the diameter of the growth zone, demonstrating that a UMH9 *cysE* mutant exhibited significantly less swarming motility than the wild-type and genetically complemented *cysE* mutant strains (Fig. 1C), as observed previously (14). The lack of swarming motility in the *cysE* mutant strain further correlated with a failure of these bacteria to differentiate into the characteristic S. marcescens swarmer morphology consisting of elongated and hyperflagellated bacterial cells (16). In comparison to the wild-type and complemented mutant strains, *cysE* bacteria exhibited shorter cell lengths and fewer flagella when collected from the leading edge of growth zones on swarm agar (Fig. 2A and B). Unexpectedly, *cysE* mutant bacteria also displayed abundant fimbriae or pili emanating from the cell surface (Fig. 2A). To investigate which products of sulfur metabolism were required for swarming, motility assays were conducted on LB agar supplemented with three organic sulfur sources (Fig. 1A). The motility of the *cysE* mutant was restored to levels similar to those of the wild-type and genetically complemented *cysE* mutant strains when swarm agar was supplemented with 1 mM cysteine (Fig. 1B and C). In the presence of either 1 mM glutathione or methionine, minimal motility was observed for the *cysE* mutant (Fig. 1B), and the resulting swarm zones were significantly smaller than those measured for both of the two control strains (Fig. 1C). For glutathione in particular, this result was unexpected, since the process of glutathione biosynthesis from cysteine is bidirectional and glutathione is known to satisfy the sulfur requirements of cysteine mutants in other bacterial species (1). Indeed, the S. marcescens *cysE* mutant had approximately 3-fold lower levels of total intracellular glutathione than wild-type bacteria (Fig. 1D). Glutathione can also serve as a source of cysteine for the S. marcescens *cysE* mutant, since the presence of 1 mM glutathione in defined M9 medium lacking amino acids allowed for wild-type levels of replication for the cysteine auxotroph (Fig. 1E). In contrast to glutathione, methionine is not expected to contribute to the pool of available cysteine in cells and does not support growth of the *cysE* mutant in defined medium (Fig. 1F). The ability of exogenous cysteine, but not glutathione, to restore swarming motility of *cysE* mutant bacteria indicates that not all sulfur sources satisfy the requirements for swarming motility and that a relative abundance of cysteine is needed.

### Transcription of hemolysin and flagellar regulon genes is influenced by products of sulfur metabolism.

**FIG 3:**
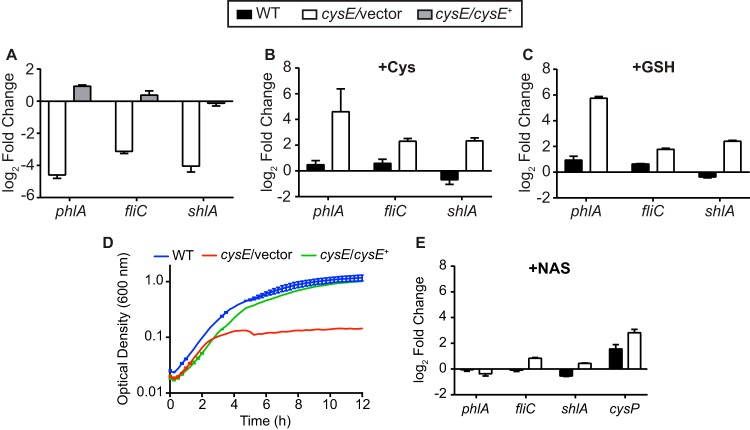
Products of sulfur metabolism contribute to expression of flagellar regulon genes. (A) Transcript levels were measured by qRT-PCR in both the *cysE* mutant (*cysE*/vector) and the complemented mutant (*cysE*/*cysE*^+^) relative to that in wild-type (WT) bacteria. All strains were cultured in M9 medium with Casamino Acids for this experiment. Transcript levels for the wild type and the *cysE* mutant strain were also determined for bacteria cultured in M9 medium containing Casamino Acids and supplemented with either 1 mM cysteine (B) or 1 mM glutathione (GSH) (C) relative to that in unsupplemented M9. (D) Wild-type, *cysE* mutant, and complemented mutant strains were cultured in M9 medium supplemented with 10 mM NAS. Growth was measured by optical density in 15-min intervals from triplicate cultures (means ± standard deviations). (E) Gene expression was determined for bacterial strains cultured in M9 medium with Casamino Acids and supplemented with 10 mM NAS relative to that in unsupplemented cultures. All qRT-PCR data are reported as the means (± standard deviations) from triplicate determinations.

Transcription of *phlA* is controlled in part by the activities of the FlhDC flagellar master regulator complex and the alternative sigma factor FliA and is coregulated with class III flagellar regulon genes of S. marcescens (17, 23). Our work further demonstrated that high levels of *phlA*, *flhD*, and *fliA* transcription are all dependent on the cysteine biosynthesis pathway (14). Based on the observation that hemolysin activity and *shlA* expression in S. marcescens is partially dependent on FlhD and FliA function (20, 21), it was predicted that *shlA* transcript levels would also be responsive to cysteine limitation. To test this prediction and establish a broader cohort of genes with which to test the influence of sulfur metabolism products, *shlA* expression levels were measured in the *cysE* mutant strain relative to that in the wild-type strain. Transcription of *shlA* was decreased 16-fold in bacteria lacking *cysE*, in agreement with expression levels for *phlA* and *fliC* (Fig. 3A). Complementation of the *cysE* mutation in *trans* resulted in levels of all three transcripts that approximated those from wild-type bacteria. The cysteine-dependent regulation of *shlA* is intriguing given that the hemolysin is considered a major S. marcescens virulence factor and is secreted via a flagellum-independent two-partner secretion mechanism (28, 29). Therefore, sulfur metabolism may influence the expression of a broader range of S. marcescens functions than initially determined and may play a role in virulence.

To test which products of sulfur metabolism were able to restore gene expression in the *cysE* mutant, relative transcript levels were compared for bacteria cultured in the presence and absence of cysteine or glutathione. As expected, providing the *cysE* mutant with exogenous cysteine increased the relative abundance of all three tested transcripts by at least 5-fold relative to that in unsupplemented cultures (Fig. 3B), while addition of cysteine to wild-type bacterial cultures resulted in little change in expression. This result is consistent with the observation that cysteine restores swarming motility of the *cysE* mutant. When *cysE* mutant cultures were supplemented with glutathione, *shlA*, *phlA*, and *fliC* all exhibited a pattern of increased expression similar to that observed for bacteria treated with cysteine (Fig. 3C). This result was surprising based on the inability of glutathione to promote swarming motility of the *cysE* mutant but indicates that glutathione can facilitate transcription of these genes when bacteria are in a cysteine-limited state. It should be noted that the swarming motility and transcriptional analyses are necessarily performed using different culture conditions and this may contribute to the inconsistent ability of glutathione to relieve cysteine limitation in these experiments.

### NAS does not induce transcription of hemolysin or phospholipase genes.

A secondary product of CysE enzyme activity is the cysteine regulon inducer NAS (Fig. 1A). The model cysteine regulons of E. coli and S. enterica are controlled by the LysR-type regulator CysB with NAS as an inducer (1). When sulfur sources are limited, NAS accumulates and activates transcription of genes associated with sulfate transport and reduction and cysteine biosynthesis. The deduced product of the S. marcescens BVG96_RS09790 open reading frame (ORF) shares 93% sequence identity with the E. coli CysB protein. Given the probable function of the CysB regulatory system in *Serratia*, it was possible that the relative lack of *phlA* and *shlA* expression in the *cysE* mutant was due to the inability of this strain to produce NAS inducer. NAS does not efficiently substitute for OAS as a precursor to cysteine biosynthesis in other organisms (1), and the S. marcescens *cysE* null mutant also grew poorly in cysteine-restricted medium supplemented with NAS (Fig. 3D). The ability of NAS to induce transcription of *phlA* and *shlA* was measured by reverse transcription-quantitative PCR (qRT-PCR) for bacteria cultured in defined medium in the presence or absence of NAS. For *shlA*, *phlA*, and *fliC*, addition of NAS to either *cysE* mutant or wild-type bacterial cultures resulted in <2-fold changes in transcript abundance compared to that in untreated bacteria (Fig. 3E). To confirm that NAS was acting as an inducer of the cysteine regulon in S. marcescens, expression of the E. coli *cysP* homolog (BVG96_RS14125, 79% amino acid identity) was also quantitated. The *cysP* gene is part of the cysteine regulon in E. coli and S. enterica and is positively regulated by CysB and NAS when bacteria are starved for sulfur (1, 4, 5, 30). NAS supplementation increased *cysP* transcription 7-fold in the *cysE* mutant strain compared to that in unsupplemented cultures (Fig. 3E). Wild-type bacteria responded to the addition of exogenous NAS by further increasing *cysP* transcription 3-fold compared to that in untreated cultures, even though the pathway for OAS and NAS production remains intact in this strain. These results are consistent with the notion that the *Serratia* cysteine regulon functions in a manner similar to that of other species. The lack of NAS-dependent regulation of *shlA*, *phlA*, and *fliC* transcription also supports the hypothesis that S. marcescens is capable of responding to cysteine limitation in a manner that extends beyond the canonical cysteine regulon.

### Sulfate levels modulate transcription of phospholipase and hemolysin genes.

**FIG 4:**
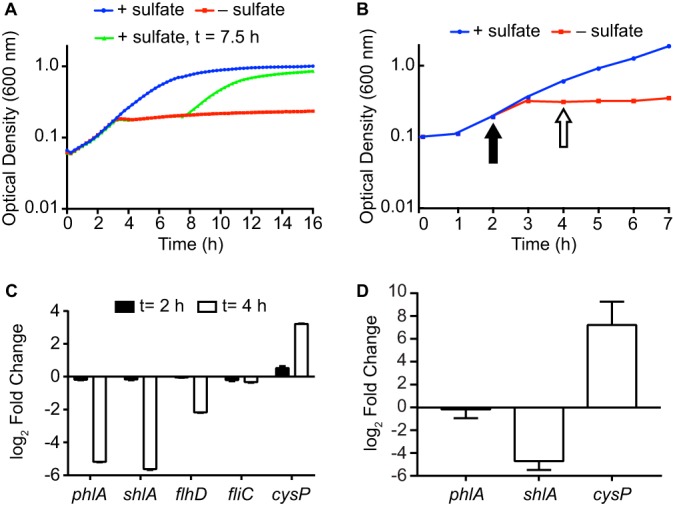
Sulfate starvation inhibits phospholipase and hemolysin gene expression. (A) The kinetics of sulfate starvation were established by culturing the wild-type strain UMH9 in sulfate-replete M9 medium (+ sulfate) or sulfate-limited medium (− sulfate). Magnesium sulfate was added after growth arrest (7.5 h postinoculation) to sulfate-limited cultures to demonstrate that sulfate was the growth-limiting nutrient under the tested conditions. (B) Strain UMH9 was cultured in sulfate-replete or sulfate-limited medium as described for panel A and used for qRT-PCR gene expression analysis. Arrows indicate the points postinoculation at which aliquots from both cultures were removed for RNA isolation and cDNA synthesis. Growth curves represent the means (± standard deviations) optical densities from triplicate cultures measured in 15-min intervals. (C) Relative expression of the indicated genes was determined by qRT-PCR for cDNA generated from the experiment in panel B. Transcript levels from sulfate-starved bacteria relative to those in sulfate-replete bacteria are presented from 2 and 4 h after inoculation. (D) Gene expression in the S. marcescens *fliP* mutant for bacteria cultured under sulfate-limited conditions relative to that for sulfate-replete bacteria. All qRT-PCR data are reported as the means (± standard deviations) from triplicate determinations.

The S. marcescens response to cysteine limitation has to this point been assessed by genetic perturbation of the cysteine biosynthetic pathway. It was therefore of interest to identify conditions under which wild-type bacteria replicated the transcriptional response observed with the *cysE* mutant. Based on the established pathway of reductive sulfate assimilation and cysteine production (Fig. 1A), it was reasoned that extracellular sulfate availability would influence transcription of phospholipase, hemolysin, and flagellar genes in *Serratia*. Before testing this prediction, sulfate starvation conditions were first established in defined media. Bacteria cultured in the absence of added sulfate grew similarly to bacteria cultured under sulfate-replete conditions until approximately 3 h postinoculation, at which point the culture density remained constant and no additional growth was observed (Fig. 4A). The observed growth kinetics indicate that sufficient sulfur (either intracellular or extracellular) is present under the tested conditions to support a limited amount of growth but that the trace sulfur is rapidly consumed, dramatically reducing replication. Addition of sulfate to sulfate-limited cultures rescued growth to levels approximating that of cultures which contained sulfate at the outset.

Using these conditions, the transcriptional profiles of bacteria in the presence and absence of sulfate, both before and after sulfate starvation, were compared (Fig. 4B). At 2 h postinoculation, there was little difference in abundance of the selected transcripts between bacteria cultured under sulfate-replete and sulfate-limited conditions (Fig. 4C). After 4 h, and at a time when bacteria had depleted the available sulfate, transcript levels for both *phlA* and *shlA* were 36- and 49-fold reduced, respectively. Expression levels of *flhD* were also reduced 5-fold at this time. These results are consistent with the observations from bacteria lacking *cysE* and cysteine and demonstrate that extracellular sulfate availability contributes to the regulation of these *Serratia* genes. Surprisingly, *fliC* levels remained consistently unchanged at both time points, indicating a difference in flagellin gene expression for the *cysE* mutant compared to that for sulfur-starved wild-type bacteria. Transcription of the S. marcescens *cysP* homolog increased 9-fold when bacteria entered sulfate starvation (Fig. 4C), consistent with the transcriptional activity of *cysP* in other bacterial species (5). These results establish that the wild-type *Serratia* strain has a dynamic response to sulfate limitation that includes increased transcription of cysteine regulon genes and a dramatic decrease in phospholipase and hemolysin genes.

To determine whether the sulfate-controlled transcription of *phlA* and *shlA* required functional flagella, expression levels were assessed in an S. marcescens *fliP* mutant lacking an essential component of the secretion apparatus. This strain was previously determined to be nonmotile in swarming assays and defective in extracellular phospholipase activity (14). Expression of *phlA* in the sulfate-starved *fliP* mutant was unchanged relative to that in bacteria from cultures containing sulfate (Fig. 4D), indicating that the modulation of *phlA* transcription via sulfate is completely dependent on flagellar function. It was anticipated that genes controlled by the *Serratia* flagellar regulatory system would be subject to FlgM-mediated sequestration of FliA in the absence of a functional flagellar secretory apparatus, based on the regulatory model of other species (31–33). In the *fliP* mutant, this could serve to diminish expression of FliA-regulated genes independently of sulfate abundance. However, both *shlA* and *cysP* expression levels were similarly responsive to sulfate starvation in the *fliP* mutant (Fig. 4D) as they were in wild-type bacteria (Fig. 4C). The requirement for flagella in sulfate-responsive expression of S. marcescens genes is therefore transcript specific, even in cases where the flagellar regulatory system contributes to gene expression, such as with *shlA* (20, 21) and *phlA*.

### Cysteine biosynthesis and sulfate availability alter hemolytic activity.

**FIG 5:**
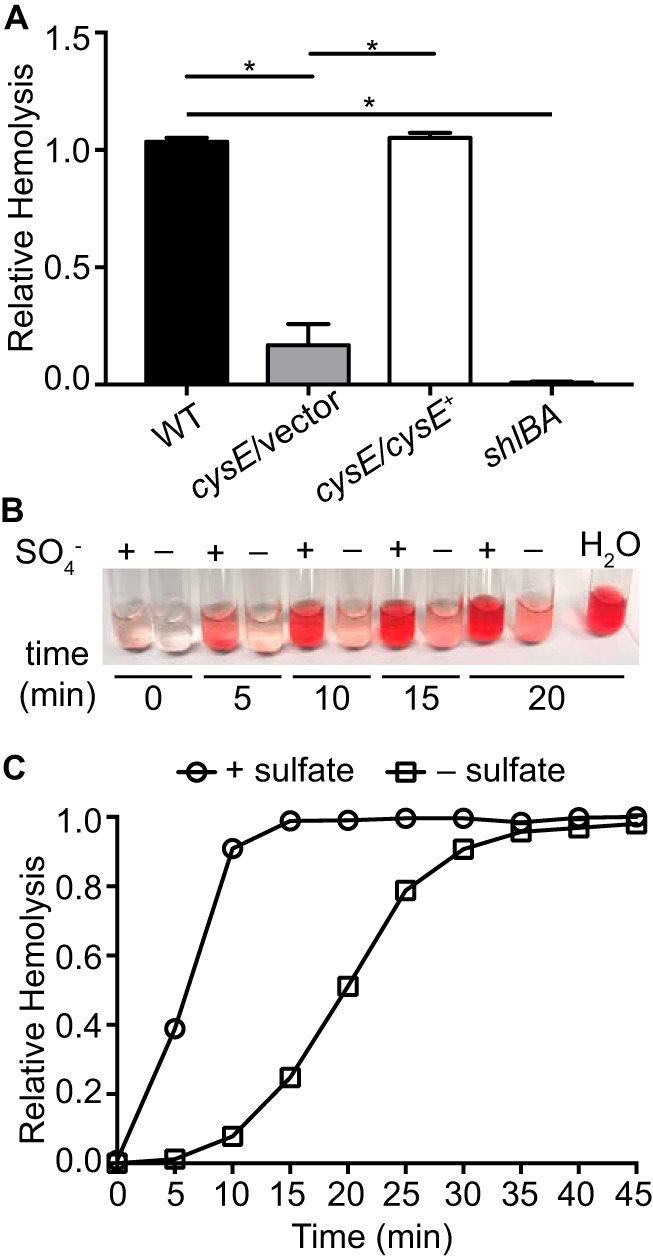
Sulfate starvation inhibits *Serratia* hemolysin activity. (A) Hemolytic activity of S. marcescens wild-type (WT), *cysE* mutant (*cysE*/vector), complemented *cysE* mutant (*cysE*/*cysE*^+^), and hemolysin null (*shlBA*) strains. Bacterial suspensions in PBS were normalized by optical density and mixed with 8% defibrinated sheep blood. Hemolytic activity was determined by measuring absorbance (405 nm) after 1 h from cleared suspensions relative to that of the positive control. Bars represent the means (± standard deviations) from triplicate assays. *, *P* < 0.001 by *t* test. (B) Representative image of a hemolytic activity assay for wild-type strain UMH9 cultured in sulfate-replete (+) and sulfate-limited (−) media. Bacterial suspensions were mixed with defibrinated sheep blood and assayed for hemolytic activity in 5-min intervals as described for panel A. (C) Quantitation of relative hemolysis over time for the wild-type strain cultured with sulfate (+ sulfate) and without sulfate (− sulfate). Results are representative of three experiments.

The targeted gene expression analyses demonstrate that the sulfur limitation response of S. marcescens has the potential to impact host interactions by modulating expression of the *shlA* hemolysin gene. Thus, it was important to determine whether the abundance of cysteine and sulfate modulated hemolytic activity of the UMH9 strain. To test this, red blood cell lysis was measured in suspension during incubation with intact bacteria. Both the wild type and the complemented *cysE* mutant strain exhibited full hemolytic activity relative to the positive control, whereas the *cysE* mutant showed a 6-fold decrease in red blood cell lysis (Fig. 5A), consistent with the transcriptional data for these strains (Fig. 3A). An *shlBA* mutant exhibited essentially no measurable hemolysis, confirming that the majority of hemolytic activity was ShlA mediated as opposed to PhlA mediated under these conditions. When wild-type bacteria were cultured in the presence of sulfate and then mixed with red blood cells, hemolytic activity proceeded very rapidly (Fig. 5B) with maximal hemolysis achieved within 10 min (Fig. 5C). In contrast, bacteria that were cultured in sulfate-limited medium exhibited considerably slower hemolytic activity. Together, these results clearly establish that sulfate and cysteine availability impact the cytolytic activity of S. marcescens and correlate well with the observed *shlA* transcriptional response.

### Sulfate-responsive transcriptome of S. marcescens.

**FIG 6:**
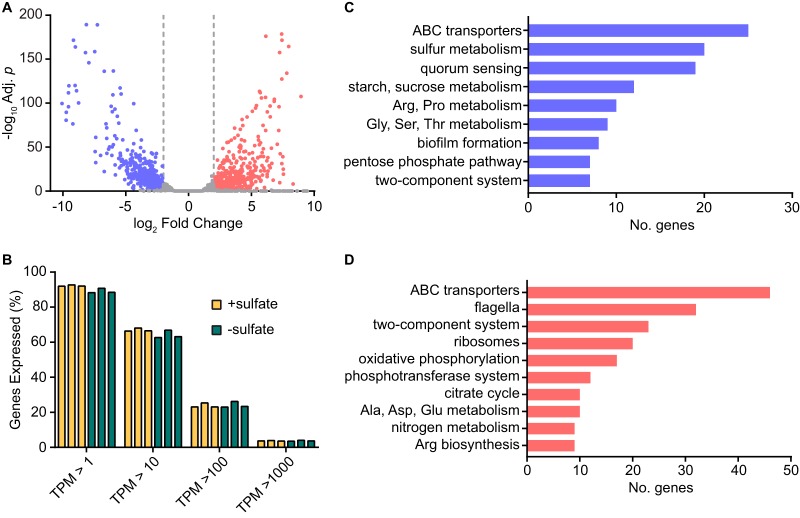
Sulfate availability alters the transcriptome of S. marcescens. Global gene expression of wild-type strain UMH9 was determined by RNA-seq for bacteria cultured in the presence of sulfate relative to that for sulfate-limited bacteria. (A) Fold change in expression was plotted for each mapped gene relative to the adjusted *P* value. A threshold of log_2_ fold change >2.0 or <−2.0 (adjusted *P* < 0.05) was used to identify significantly upregulated (red) and downregulated (blue) genes. (B) The proportions of total UMH9 genes having transcripts per million (TPM) values between 1 and 1,000 under the two tested conditions. (C and D) Protein-encoding genes were annotated by KEGG BlastKOALA to identify the top biological pathways affected by sulfate availability. Upregulated and downregulated pathways are colored as described for panel A.

The diverse set of genes thus far identified to be regulated by cysteine metabolism functions prompted an investigation into the global sulfur transcriptional response of S. marcescens. The complete repertoire of S. marcescens genes that are altered by sulfate availability was determined by transcriptome sequencing (RNA-seq) analysis using the wild-type UMH9 strain. The expression profiles of bacteria cultured in sulfate-replete and sulfate-limited media were compared, yielding a remarkable total of 1,130 genes that exhibited significantly altered transcript levels under these conditions (log_2_ fold change > 2.0 or <−2.0, adjusted *P* < 0.05) (see Data Set S1 in the supplemental material). Within this cohort of genes, there was an even distribution of differentially expressed transcripts, with 575 more highly expressed in the presence of sulfate and 555 more highly expressed in the absence of sulfate (Fig. 6A). These results demonstrated that the sulfur response is a dynamic process that dramatically shifts the transcriptional profile of S. marcescens. Despite the shift in abundance for these specific transcripts, the overall distributions of gene expression across the range of high- and low-abundance transcripts remained similar between the two tested conditions, which serves as a validation of the transcriptomic comparison (Fig. 6B).

10.1128/mSystems.00285-19.1 DATA SET S1 Differentially expressed genes of S. marcescens UMH9 cultured in sulfate-replete and sulfate-limited media. Significant changes in genes expression were defined as having a log_2_ fold change of >2.0 or <−2.0 (adjusted *P* < 0.05). Download Data Set S1, XLSX file, 0.2 MB. Copyright © 2019 Anderson et al. 2019 Anderson et al. This content is distributed under the terms of the Creative Commons Attribution 4.0 International license.

**FIG 7:**
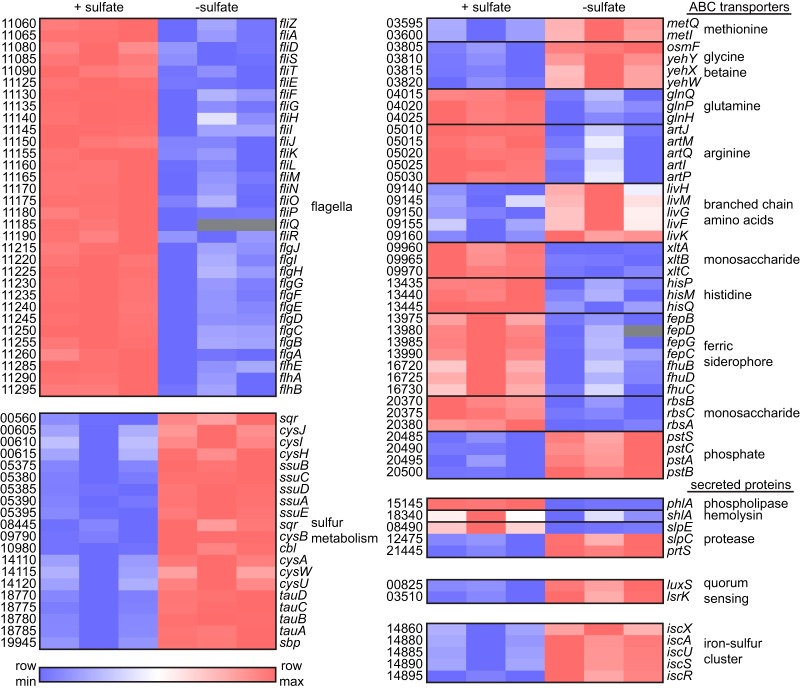
Selected S. marcescens transcripts that are differentially regulated by sulfate availability. Relative expression of individual genes as determined by RNA-seq for triplicate cultures of UMH9 in sulfate-replete (+ sulfate) and sulfate-limited (− sulfate) media. Putative gene names were assigned based on homology to genes of known function in other bacterial species, and numbers designate open reading frames according to the NCBI UMH9 reference sequence annotation (e.g., BVG96_RS11060, *fliZ*). The heat map was generated using log_2_-transformed TPM values. Gray boxes indicate missing values.

A characterization of the differentially expressed protein-encoding genes was conducted using KEGG pathway analysis (34) to gain insight into the physiologic response of S. marcescens to sulfur. There are at least 28 established cysteine biosynthesis and sulfur metabolism genes that comprise the cysteine regulon of E. coli (1). Of these, 20 S. marcescens UMH9 homologs were identified as having significantly elevated transcription under sulfate-limited conditions (Fig. 6C). Included within this group were genes encoding proteins for transport of sulfur sources, sulfate assimilation pathway proteins, and transcriptional regulators, together providing strong evidence that the cysteine regulon of E. coli is largely conserved in S. marcescens (Fig. 7). Additionally, genes for five ISC-type iron-sulfur cluster assembly homologs (35) were significantly elevated during sulfate limitation, possibly reflecting a deficit in cluster formation when sulfur is lacking. Another functional category with a high representation of genes with increased expression during sulfate limitation included S. marcescens homologs of bacterial genes related to quorum sensing (Fig. 6C). Notably, the *luxS* gene associated with autoinducer-2 production in S. marcescens (36) and a gene encoding an LsrK autoinducer-2 kinase homolog exhibited 8-fold and 5-fold increases, respectively, in expression during sulfate limitation (Fig. 7). Whether quorum sensing functions are increased under sulfur-limiting conditions remains to be determined for strain UMH9, but this observation serves to illustrate that a diverse range of functions are responsive to sulfate starvation and have the potential to initiate a major shift in the physiology of bacteria in sulfur-limited environments.

Based on the quantitative qRT-PCR data for *flhD* (Fig. 4C) and results obtained with the *cysE* mutant, it was expected that expression of S. marcescens flagellar genes would be elevated in the transcriptomic analysis when bacteria were cultured with abundant sulfate. Thirty-two flagellar genes were significantly upregulated under this condition compared to that in sulfate-limited bacteria, confirming the importance of sulfur availability for flagellar function (Fig. 6D and 7). Fimbria or pilus expression has been shown to be inversely correlated with the production of flagella and motility in several bacterial species (37–40). Thus, the observation that the swarming-deficient *cysE* mutant produced abundant cell surface fimbriae (Fig. 2A) prompted an exploration of putative fimbrial genes that were differentially expressed under the tested conditions. In total, 11 predicted fimbria-related genes were differentially regulated (Data Set S1). Among these, four genes clustered to an uncharacterized chaperone-usher fimbria locus that was significantly upregulated during sulfate limitation, potentially corresponding to the fimbria production observed with the *cysE* mutant. The seven remaining genes identified within this functional group were all more highly expressed in the presence of sulfate (Data Set S1).

The *phlA* phospholipase and *shlA* hemolysin genes were significantly upregulated in the presence of sulfate as measured by RNA-seq, in agreement with the qRT-PCR results (Fig. 7). S. marcescens is known to secrete a number of additional proteins, including chitinases, a nuclease, and metalloproteases of the serralysin family that have been implicated in virulence (41–43). Of the four predicted serralysin-type proteases encoded in the UMH9 genome, three were differentially regulated under the tested conditions, but only the *slpE* homolog shared a similar expression pattern to those of *phlA* and *shlA*. The three predicted extracellular chitinases and extracellular nuclease of UMH9 were not differentially regulated by sulfate availability (data not shown).

The annotation group with the greatest number of differentially expressed genes under both conditions was ABC transporters (Fig. 6C and D). A total of 76 genes encoding components of 30 different predicted ABC transport complexes were identified. The predicted substrates for these transport complexes included sugars, amino acids, and ions. Both methionine and branched-chain amino acid transporters exhibited increased expression in the absence of sulfate, whereas glutamine, arginine, and histidine transporters were more highly expressed in the presence of sulfate (Fig. 7). This differential response of transporter expression may reflect an energetic advantage to transport rather than synthesis of certain amino acids in environments where sulfur sources are abundant or, in some cases, may be a consequence of a condition-specific ability to synthesize amino acids, such as methionine. Notable among the ion ABC transporters were two systems associated with catecholate and hydroxamate ferric siderophore utilization. These, together with several predicted TonB-dependent siderophore receptors (Data Set S1), exhibited significantly higher expression levels in the presence of sulfate (Fig. 7). This result is intriguing considering that iron levels were not manipulated between the two tested culture conditions and the important role that iron acquisition systems play in bacterial survival in the iron-restricted environment of the mammalian host (44).

## DISCUSSION

We have defined the sulfur regulon of S. marcescens using a transcriptomic approach to identify differentially regulated genes in the presence and absence of sulfate. This regulon includes numerous genes that encode products of sulfur homeostasis, for example, those involved in cysteine biosynthesis, sulfate transport and reduction, and iron-sulfur cluster assembly. Such a response to sulfur limitation was anticipated and appears to be conserved in diverse bacterial species (1, 45–47). Importantly, we have also identified a large cohort of S. marcescens transcripts, elevated in either the presence or absence of sulfur, that have no obvious function in sulfur acquisition and utilization. Thus, the S. marcescens response to sulfate abundance represents a dramatic shift in bacterial physiology that includes differential regulation of putative virulence factors, nutrient transport functions, and metabolism.

The FlhDC and FliA regulatory system that controls hierarchical transcription of genes required for function of the flagellar export and motility apparatus has been extensively studied in multiple bacterial species (48, 49). This system responds to internal and external cues to control flagellar production and motility and, for *Serratia*, also regulates transcription of genes encoding the extracellular phospholipase and hemolysin (14, 17, 20, 21, 23). The *Serratia* flagellar regulatory system is modulated by multiple signals, including temperature, enterobacterial common antigen production, cAMP, and fatty acids, (19, 20, 22, 50–52). Together with our previous work, the transcriptional profile reported here establishes that sulfur availability represents an additional signal for flagellar function in S. marcescens. The coregulation of *Serratia* phospholipase and flagellar components is not surprising in light of data indicating that secretion of the PhlA enzyme is mediated by the flagellar apparatus (17, 18). The biological significance of hemolysin gene control via the flagellar regulatory system is currently less well understood. However, the sulfur-dependent coregulation of these genes suggests that flagella, phospholipase, and hemolysin may serve beneficial functions within similar sulfur-containing environments. Extracellular phospholipases and flagella contribute to pathogenesis in other bacterial species, though a conclusive role for these proposed virulence factors remains to be determined for S. marcescens. The S. marcescens hemolysin is, however, an established virulence factor in both cell-based and murine models of infection (20, 25–27, 53). Given the potential virulence implications for some of the sulfur-dependent genes identified in this study, it is important to consider the levels of available sulfur at relevant sites of infection in the mammalian host. Healthy adult human serum is estimated to contain >0.2 mM sulfate anion and 60 to 120 μM cystine (54). Adult urine also contains abundant sulfate (>2 mmol/mmol creatinine) but lower levels of cystine (2 to 50 μmol/mmol creatinine) (54). Based on these examples, we speculate that the host environment contains sufficient sulfur sources to allow for the efficient expression of S. marcescens sulfur-dependent genes. It should also be noted that S. marcescens may conversely benefit from decreased expression of sulfur-dependent virulence factors and other genes when sulfur sources are limited. Further investigation will be required to determine which of the diverse niches of S. marcescens represents such a condition, since the concentration of sulfur, in its many organic and inorganic forms, varies greatly within the environment.

It is likely that the conserved sulfur assimilation regulators CysB and Cbl play a role in the S. marcescens transcriptional response to sulfate (Fig. 7). However, given the number of genes whose expression is altered by the availability of sulfate and the diversity of functions encoded by these genes, it is anticipated that multiple regulatory processes contribute to this response. In the absence of serine acetyltransferase activity, neither OAS nor NAS is produced; therefore, CysB is not induced during sulfur limitation. A lack of induction by NAS could explain the relative decreases in *phlA* and *shlA* transcript levels in the S. marcescens *cysE* mutant compared to those in wild-type bacteria if these genes were part of the cysteine regulon. However, a relative increase in CysB-dependent genes would be expected when *cysE* mutants are provided with exogenous inducer, as reported previously (4) and observed here with *Serratia cysP* (Fig. 3E). The lack of induced *shlA* or *phlA* expression under these conditions suggests that the hemolysin and phospholipase genes are independent of the cysteine regulon. This conclusion is further supported by our observation that cysteine alone can restore the loss of *phlA* and *shlA* transcripts in *cysE* mutant bacteria (Fig. 3B). Importantly, the presence of exogenous cysteine overcomes the auxotrophy of *cysE* mutants but is not expected to provide a source of NAS inducer. These data support the conclusion that cysteine or a cysteine-dependent function is important for transcriptional control of *shlA* and *phlA* rather than the presence of NAS inducer. The requirement for cysteine in the transcription of these and other sulfate-regulated genes is potentially indirect and could be accomplished by multiple means. One possibility is that sulfate and cysteine limitation may disrupt bacterial redox homeostasis by altering glutathione concentrations. Consistent with this notion, E. coli mutants that are defective in either glutathione synthesis (*gshA*) or glutathione export across the cytoplasmic membrane (*cydD*) have altered periplasmic redox states, which result in swarming motility defects that can be rescued by exogenous cysteine or glutathione (55–57). Furthermore, *cydD* mutants exhibit pleiotropic changes in transcription compared to wild-type cells (58). The decreased intracellular glutathione concentrations observed in the *Serratia cysE* mutant indicate that glutathione synthesis is disrupted when cysteine is limiting and are consistent with the swarming motility defects of E. coli *gshA* and *cydD* mutants. It is therefore possible that dysregulated flagellar function following thiol-dependent redox perturbation could alter the transcription of FlhDC-dependent and FliA-dependent genes. We have observed here that the swarming motility defect of a *Serratia* cysteine auxotroph can be restored by cysteine but not glutathione, despite the ability of glutathione to restore expression of flagellum-associated genes in this strain. The reason for this discrepancy is not currently known, although it is possible that the different experimental conditions employed may influence the balance of reduced glutathione and oxidized glutathione disulfide in each assay.

In summary, our results demonstrate that flagella, phospholipase, and hemolysin functions of S. marcescens are all modulated by the sulfur assimilation process. Furthermore, the genes encoding these functions are members of the sulfate regulon of S. marcescens, which was defined in this work as encompassing >1,000 genes. This global response to sulfur availability is likely important for adaptation of S. marcescens to the many environmental niches occupied by this organism.

## MATERIALS AND METHODS

### Bacterial strains and culture conditions.

S. marcescens UMH9 is a clinical isolate from a patient with bacteremia and was described previously (59). The UMH9 *cysE* and *fliP* mutants were also described previously (14). Genetic complementation of the *cysE* mutation was accomplished by expression of *cysE* from a previously described plasmid (14), with the parent plasmid pBBR1MCS-5 (60) serving as the vector control. E. coli TOP10 (Thermo Fisher Scientific) cells were used for routine cloning purposes. Bacteria were cultured in LB medium (61) unless indicated otherwise. Growth of S. marcescens strains under defined conditions was accomplished using M9 medium (62). The M9 base medium contained 0.4% glycerol as a carbon source, and additional supplements were added where indicated. Experiments involving growth of the *cysE* mutant in M9 medium required the addition of 0.1% Casamino Acids to all cultures, with the exception of experiments testing the utilization of specific sulfur sources. The M9 base medium contained 1 mM MgSO_4_ and was considered sulfate replete. Sulfate-limited conditions were established by substituting 1 mM MgCl_2_ for MgSO_4_. Glutathione and l-cysteine were prepared as aqueous solutions from each compound in their reduced states and used immediately. When required, selective antibiotics were added to culture media at the following concentrations: gentamicin, 6 μg/ml; kanamycin, 50 μg/ml; spectinomycin, 100 μg/ml.

### Mutant construction.

The S. marcescens *shlBA* mutant was constructed by lambda Red recombineering (63). Oligonucleotide primers (see Table S1 in the supplemental material) containing ∼50 bp of target site homology at the 5′ ends were used to amplify the kanamycin resistance gene from pKD4 (64). The resulting PCR fragment was introduced by electroporation into strain UMH9 harboring the recombineering plasmid pSIM19 (65). The Δ*shlBA*::*nptII* mutation was confirmed via sequencing, and pSIM19 was cured from the strain using established methods (63).

10.1128/mSystems.00285-19.2 TABLE S1 Oligonucleotide primers used in this study. Download Table S1, DOCX file, 0.01 MB. Copyright © 2019 Anderson et al. 2019 Anderson et al. This content is distributed under the terms of the Creative Commons Attribution 4.0 International license.

### Growth assays.

S. marcescens strains were cultured overnight in LB medium and then washed and subcultured into M9 medium supplemented with 1 mM glutathione, 1 mM methionine, or 10 mM NAS. Subcultures were inoculated at a calculated optical density (600 nm) of 0.01 in 100-well microplates. Growth was assessed by optical density using a Bioscreen C instrument at 37°C with readings taken in 15-min intervals. For sulfate starvation analysis, bacteria from sulfate-replete M9 overnight cultures were washed and subcultured in sulfate-replete or sulfate-limited medium at a calculated optical density of 0.1. Magnesium sulfate was added to sulfate-limited cultures 7.5 h after inoculation, where indicated. Growth was measured with the Bioscreen C apparatus as described above.

### Motility assay.

Swarming motility was assessed using LB medium solidified with 0.6% agar and supplemented with cysteine, glutathione, or methionine at a final concentration of 1 mM where indicated. Bacteria cultured overnight in LB liquid medium were used to inoculate (10 μl) the surfaces of swarm plates. After 16 h of incubation at 30°C, the swarm zone diameter was measured and images were taken using a Q-count automated colony counter (Spiral Biotech).

### Hemolytic activity assay.

Extracellular hemolytic activity was measured by lysis of sheep red blood cells based on previously reported methods (21). Briefly, S. marcescens strains were cultured overnight in M9 medium with Casamino Acids at 30°C, collected by centrifugation, and resuspended in phosphate-buffered saline (PBS) to an optical density of 0.7. Bacterial suspensions were mixed in equal volumes with 8% (vol/vol) defibrinated sheep blood and incubated at 30°C for 1 h. Intact red blood cells and bacteria were pelleted by centrifugation, and the absorbance at 405 nm was used to measure the amount of released hemoglobin. For the assessment of hemolytic activity over time from bacteria cultured under sulfate-replete and sulfate-limited conditions, strain UMH9 was resuspended to an optical density of 0.4 before mixing with defibrinated blood. Suspensions were incubated at 30°C, and the absorbance of cleared samples was measured every 5 min for a total of 30 min. Complete lysis and nonspecific lysis were determined by incubating 8% defibrinated sheep blood with water and PBS, respectively. Hemolytic activity was calculated relative to the water-containing positive control.

### Microscopy.

The morphology of *Serratia* strains cultured under swarming conditions was determined by both transmission electron microscopy and light microscopy following flagellar stain. For electron microscopy, bacteria from the leading edges of swarming populations were collected with an inoculation loop and transferred to a 20-μl drop of water. Copper grids (300 mesh; Ted Pella) were applied to the water drops to allow for adhesion of bacterial cells. Grids were stained with 1% phosphotungstic acid for 1 min and rinsed briefly with water prior to analysis. Images were acquired at the University of Michigan Microscopy and Image Analysis Laboratory with a JEOL JEM-1400 transmission electron microscope (TEM) using an accelerating voltage of 80 kV. Bacteria from the periphery of the swarm zones were also visualized by light microscopy following flagellar staining according to the manufacturer’s instructions (Hardy Diagnostics). Images were acquired on an Olympic BX60 microscope using a 100× lens objective.

### Quantification of intracellular glutathione.

Intracellular glutathione levels were measured using a commercially available kit (Sigma-Aldrich). S. marcescens strains were cultured in LB medium for 6 h prior to conducting the assay. Bacterial lysates were deproteinized and the assay was performed according to the manufacturer’s recommendations. Purified glutathione was used to generate a standard curve, and the total intracellular glutathione was normalized to the optical density of each culture.

### qRT-PCR.

Bacteria were cultured overnight in M9 medium or M9 medium containing Casamino Acids in the case of experiments involving the *cysE* mutant. Bacteria were then subcultured in the same medium with or without supplementation of cysteine, glutathione, NAS, or sulfate prior to harvesting bacteria for RNA isolation. Bacteria were stabilized with RNA Protect reagent (Qiagen) at the time of harvest, and synthesis of cDNA was performed as described previously (14). Oligonucleotide primers that were used to amplify internal fragments of the *phlA*, *shlA*, *fliC*, *flhD*, *cysP*, and *gyrB* genes are listed in Table S1. Amplification data were acquired using a QuantStudio 3 Real-Time PCR system (Thermo Fisher), and relative gene expression was determined via the comparative cycle threshold (*C_T_*) method (66).

### Transcriptomics.

The wild-type UMH9 strain was cultured in sulfate-replete M9 medium overnight, and bacteria were washed with M9 salts before subculturing in sulfate-replete and sulfate-limited M9 media. Cultures were incubated for 4 h at 37°C, at which point, bacteria were harvested and RNA was isolated. DNase-treated total RNA was used to generate sequencing libraries using an Illumina ScriptSeq kit. Library preparation and sequencing of cDNA libraries on an Illumina HiSeq 2500 platform were performed by the University of Michigan DNA Sequencing Core. Raw fastq files were processed with Trimmomatic (v. 0.36) (67) to remove adapter sequences and analyzed with FastQC to check for poor quality samples. Mapping was performed with Bowtie 2 aligner (v. 2.3.4) (68) using default parameters. Read counts were calculated using HTseq (v. 0.9.1) htseq-count (union mode) (69). Differential expression analysis was performed using DESeq2 R package (70). Genes with log_2_ fold change of >2 or <−2 and an adjusted *P* value (Benjamini-Hochberg adjustment) of less than 0.05 were considered to be differentially expressed. Pathway analysis was performed on protein-encoding genes using the KEGG BlastKOALA function (71).

### Data availability.

cDNA sequence reads from the RNA-seq experiments have been deposited in the National Center for Biotechnology Information Sequence Read Archive under accession PRJNA535513.
