# Supplementary material for: Sulfur Assimilation Alters Flagellar Function and Modulates the Gene Expression Landscape of Serratia marcescens
Source: mSystems. 2019 Aug 6;4(4):e00285-19. doi: 10.1128/mSystems.00285-19 (PMC6687942; doi:10.1128/mSystems.00285-19)
Supplement: TABLE S1 [file mSystems.00285-19-st001.docx]

| **Primer name** | **Sequence (5ʹ - 3ʹ)** |
| --- | --- |
| Recombineering |  |
| Δ*shlBA::*kmF | GCCCTTTCCGCCGAAACCCTGCCGGACGCCCACATGATGCGGGATGTGTAGGCTGGAGCTGCTTC |
| Δ*shlBA::*kmR | GGCTTTGGCGTCCACCTGTTTTGTTGAGGCATGGCCGCTGACGAATGGTCCATATGAATATCCTCCTTAGTTCC |
| qRT-PCR |  |
| *phlA*Fq | GTTTCCAGGCCGGGATTTA |
| *phlA*Rq | TGTACTGCACATCGTCATAGC |
| *fliC*Fq | GAGAAAGAAGTGGCTCCTACTG |
| *fliC*Rq | GTCTACGGTCGCCTTGAAATA |
| *flhD*Fq | CGATGTTCCGTCTTGGTATTGA |
| *flhD*Rq | GTTAAAGCGGAAGTGGCAAAC |
| *shlA*Fq | GGCGCATCAACATCATCAAC |
| *shlA*Rq | CGAATATCGTAGGCTTTCACCT |
| *cysP*Fq | GCCAAAGCCTATCTGAACTATCT |
| *cysP*Rq | CCATCGCTTTCTGGTCATACA |
| *gyrB*Fq | CTCTTCTCAGACCAAGGACAAG |
| *gyrB*Rq | CCGGATTCTCCATCAGGTAATC |
